# Two Cases of Dextromethorphan Overdose Reversed by Naloxone

**DOI:** 10.7759/cureus.34501

**Published:** 2023-02-01

**Authors:** Dhruva Kumar, Varsha Shinde, Sweta Khuraijam, Anjeeth Puthoor

**Affiliations:** 1 Emergency Medicine, Dr. Dnyandeo Yashwantrao (D. Y. Patil Medical College, Hospital & Research Centre, Pune, IND; 2 Emergency Medicine, Shija Hospitals and Research Institute, Manipur, IND

**Keywords:** india, toxicology, naloxone, young adults, substance abuse, dextromethorphan

## Abstract

We present two rare cases highlighting the rare toxicological manifestation of dextromethorphan (DXM). The DXM toxicity profile is predominantly hallucinations, agitation, irritability with seizures, and coma in severe overdose. The cases that follow are unique in the sense that both patients had features of opioid toxidrome, rarely manifested in DXM abuse.

A young male and female in their mid-20s and early 30s, respectively, were brought to the emergency room for their excessive somnolence; both had reduced respiratory rate, bilaterally small pupils (sluggish reactive to light), and the rest of their examination findings were unremarkable. Primary stabilization in the form of noninvasive ventilation (NIV) trial and subsequent rapid sequence intubation (RSI) for persistent respiratory depression. Followed by the exhaustive exclusion of differentials, opioid-like toxidrome was treated with naloxone, and both patients made a good recovery and were discharged home in good health.

The emergency physician should be prepared for the rare toxicological manifestations of commonly available over-the-counter medications among the youth. These case reports highlight the role of naloxone in DXM toxicity reversal.

## Introduction

The COVID-19 pandemic was a wake-up call to the world population as they contemplated the joys of life outside confined quarters, pensive about when they can get back to living the semblance of living a regular life. Around the same time, restlessness and the desire to pander to their urges of abuse led a section of the population to experiment with the more easily available legal highs, namely over-the-counter medication.

Self-medication is a global problem but one that can have troubling consequences in a nation as big in its population as India. A doctor-to-patient ratio of 1:854 falls well below the advised 1:1000 [1]. Patients of the lower strata in the Indian population, of which there is a strong number, cannot afford healthcare, and that lends its hands to the above concern. When medications such as cough syrups contain drugs that can induce hallucinations and dissociative symptoms when taken at supratherapeutic doses are left with the public to abuse, it is but a matter of time till the whole problem takes on an entire movement in and off itself.

The abuse of antitussives noted since the early 1990s has seen a waxing and waning trend globally due to multifactorial causes; during those times, the content was codeine-based antitussives, and since its ban, a newer and safer drug was presumed to have been released to the general public as an alternate: dextromethorphan (DXM) [2]. The abuse of DXM saw an insidious peak and, after receiving media attention, plateaued by 2006 and, by 2015, has taken a substantial dip as per the Food and Drug Administration (FDA) advisory committee [3].

The DXM toxicological profile classically is a mix of ketamine-like dissociative states and phencyclidine profile mixture, i.e., agitation, restlessness, hyperthermia, hallucinations, paranoia, with reduced respiratory effort and seizure, and coma in the severely overdosed individuals. No antidote is cited for DXM overdose, while rare case reports record complete reversal of DXM overdose by opioid antagonist naloxone. The below cases discuss regarding the rare manifestation of opioid toxidrome in a DXM overdose and their effectiveness and reversal using naloxone [4-7]. 

## Case presentation

Two young adults, a 20-year-old male student and a married woman in her mid-30s, were brought to the emergency room by their attenders for complaints of excessive somnolence and reduced responsiveness.

Both were nonambulatory and were stretchered into the emergency room (ER); both the individuals had a threatened airway and reduced respiratory effort (7c/min and 6c/min, respectively), hypoxic (90% and 86%, respectively) with stable circulatory vitals. The male patient was drowsy and arousable (Glasgow coma scale (GCS) of 12/15), and the female was only arousable on painful stimuli (GCS 9/15) with pinpoint pupils that were non-reactive to light. Both patients were propped up and started on oxygen therapy via face mask; the nasopharyngeal airway was secured, and both patients were given a non-invasive ventilation (NIV) trial in view of type 2 respiratory failure with reduced respiratory rate, arterial blood gas (ABG) (Table 1). No obvious findings were noted on exposure. Bedside glucose test was blood glucose test revealed 119mg/dl for the male and 96mg/dl for the female patients, respectively (<60 - >200mg/dl). Both of the patients were subject to a point-of-care arterial blood gas (ABG), X-ray chest, and COVID-19 rapid antigen.

**Table 1:** Contains ABG values for both the patients suggestive of type 2 respiratory failure, the male patient, in addition, had features of lactatic acidosis with hyperkalemia ABG - arterial blood gas

Patient	Male	Female	Normal
pH	7.09	7.1	7.35-7.45
pCO^2^ (mmHg)	66	58	35-45
pO^2^ (mmHg)	95	99	75-100
K^+ ^(mEq/L)	6.6	5	3.5-5.2
Lac	3	1.9	1.2-2.2
Bicarbonate (mEq/L)	20.3	21	22-26
FiO^2 ^(%)	60%	40%	21%

**Figure 1:**
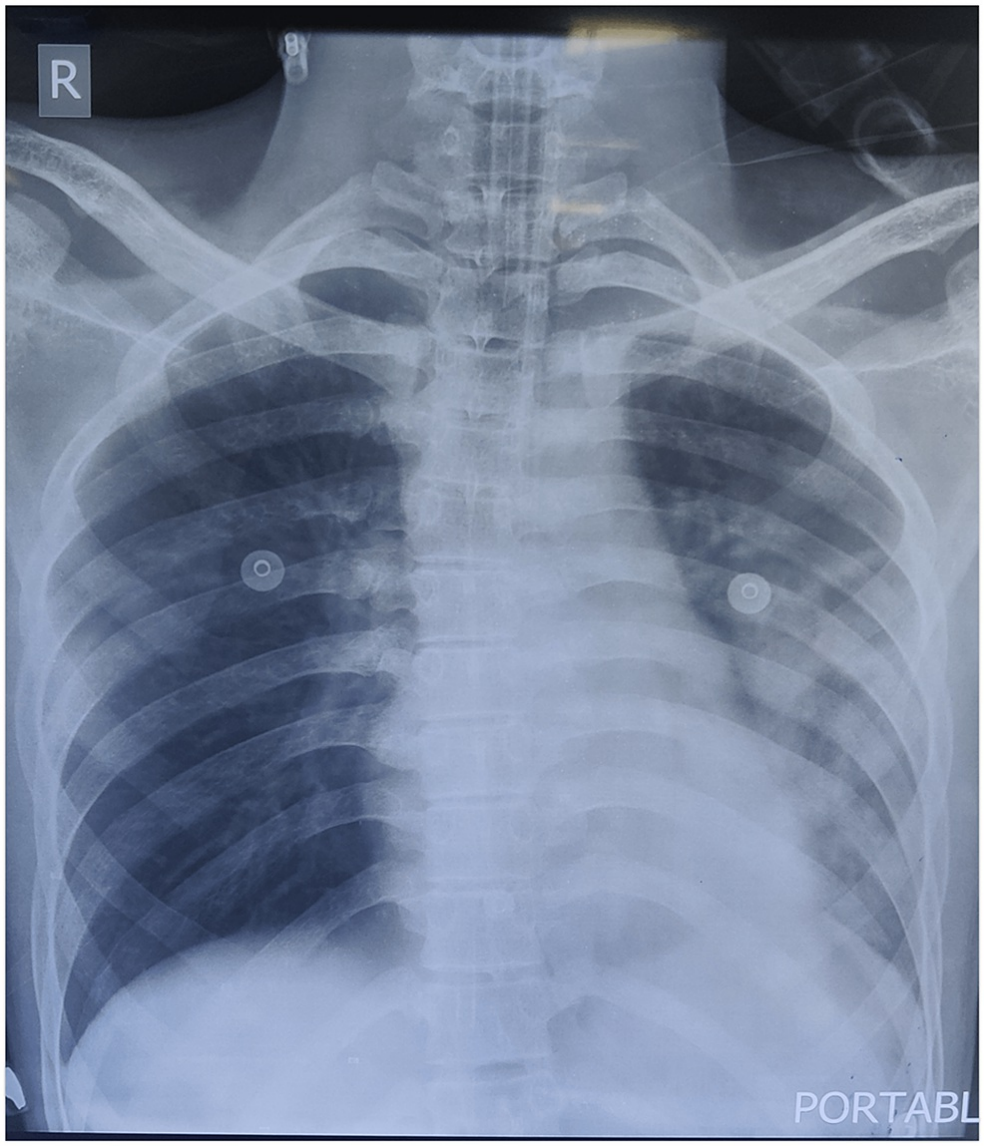
Portable X-ray chest of the male patient with homogenous opacities visualized in the left lung involving the lower lobe predominantly

The arterial blood gases revealed acute respiratory acidosis for both. X-ray of the male patient showed consolidative changes in the left middle to lower zone (Figure 1). The patients were both intubated under repetitive strain injury (RSI) due to the inability to maintain a patent airway and failure of NIV.

Differentials ranging from occult neuroparalytic snake bite, alcohol intoxication, sepsis, electrolyte imbalance, cerebrovascular accident (pontine hemorrhage), metabolic encephalopathy, and opioid abuse were considered, and appropriate investigations and labs were sent.

The male's bloodwork revealed hyperkalemia, deranged renal function tests, and leukocytosis and he was treated expediently (Table 2). 

**Table 2:** Displaying the bloodwork of the male patient with leukocytosis, deranged renal function tests, and hyperkalemia SGOT - serum glutamic-oxaloacetic transaminase, SGPT - serum glutamic pyruvic transaminase, ALP - alkaline phosphatase

Blood work up	Patient	Normal
Haemoglobin	10g/dl	13.2-16.6g/dl
Total leukocyte count	12,000/micL	4000-10,000/micL
Platelets	2/micL	1,50,000-4,10,000/micL
Urea	37mg/dl	17-49mg/dl
Creatinine	1.75mg/dl	0.6-1.35mg/dl
Sodium	133mmol/l	136-145mmol/l
Potassium	6mmol/l	3.5-5.1mmol/l
Chloride	104mmol/l	98-107mmol/l
Total bilirubin	0.2mg/dl	0.22-1.2mg/dl
Direct bilirubin	0.16mg/dl	<0.5mg/dl
SGOT	60U/l	8-48U/l
SGPT	52U/l	7-55U/l
ALP	90U/l	55-149U/l

**Figure 2:**
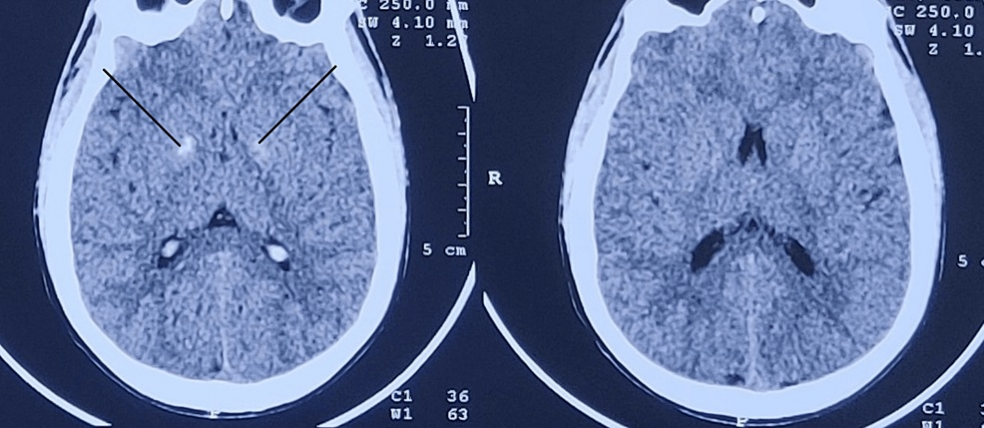
CT brain plain of the male patient showing b/l basal ganglia calcifications, noted in individuals with chronic substance abuse, marked by two arrows

**Figure 3:**
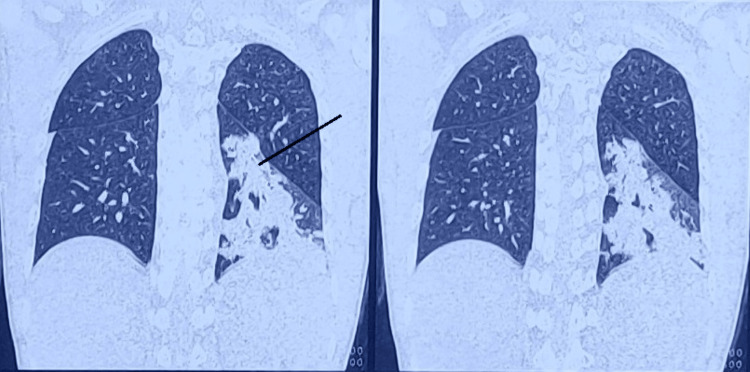
HRCT thorax suggestive of left lower lobe consolidation with minimal effusion, aspiration pneumonitis, marked by an arrow HRCT - high-resolution computed tomography

Radiological investigations of the male, including the high-resolution computed tomography (HRCT)-thorax and computed tomography (CT)-brain were suggestive of left lower lobe consolidation aspiration pneumonitis and b/l basal ganglia calcifications, respectively (Figure 2, 3). 

Due to an incomplete understanding of the clinical condition, the patient's relatives and friends were further questioned regarding the possibility of any substance abuse. It was then brought to light regarding the patient's abuse of the cough syrup bottles noticed over the past six months. Both of the patients were alleged to have consumed more than two bottles of cough syrup containing dextromethorphan and chlorpheniramine maleate the night prior.

The evidence of cough syrup abuse led the treating physician to administer naloxone; the patients both had a drastic improvement in their mentation along with respiratory drive. Both of the patients were administered multiple boluses of naloxone, followed by transfer to the medical ICU, where both were soon extubated and, within the week of admission, discharged home in good health and with psychiatric counseling.

## Discussion

Early resuscitation and acknowledging the incomplete clinical picture resulted in the diagnosis and definitive treatment of the ailment. The use of CT-brain and extensive bloodwork could have been avoided had the information regarding the cough syrup abuse been mentioned earlier during history taking. The patients might have been spared intubation and the associated discomfort.

DXM is a readily available and a codeine analog hence its introduction into the formulations of cough syrup in codeine's stead since its ban; DXM is a drug very similar in its chemical structure to the other opioids but has minimal opioid receptor activity. Despite having insignificant activity among the opioid receptors, DXM crosses the blood-brain barrier and acts upon the sigma opioid receptor leading to the suppression of cough. In overdose scenarios, the concomitant activity on the N-methyl-D-aspartic acid (NMDA) receptors leads to the effects like hallucinations and euphoria. At higher doses may lead to agitation, muscle twitches, and seizures and might result in a coma. With no antidote or specific treatment guidelines to outline the plan of treatment in acute toxic ingestion of the drug, the management is supportive, although reports of individuals treated with naloxone reversed the toxic effects of DXM [8].

Substance abuse pertains to the usage of a drug in such amounts that it becomes harmful to the user in question. Commonly abused substances can be broadly classified into opioids (heroin, morphine), stimulants (cocaine, MDMA), hypnotics (benzodiazepines, barbiturates), hallucinogenic (lysergic acid diethylamide (LSD), phencyclidine), etc. DXM, although a hallucinogenic, as mentioned previously, is a well-known alternative to the above more illicit and potentially lethal vices [9].

Bryner et al. have concluded in their study that the majority of the population in their study with problems pertaining to abuse of cough syrup containing DXM belonged to the nine to 17 years of age; unfortunately, in India, the demography cannot be accurately narrowed down since there have not been enough cases documented. This legal high needs to be addressed by the media and legislative policies to help the vulnerable public avoid falling into the chasm of addiction and substance abuse [10].

## Conclusions

Emphasis on a thorough physical examination and detailed history taking can reduce the time to definitive therapy and avoid morbidity, and in rare instances, mortality for the patients concerned, especially during emergency situations. We would like to advocate the advantage of using naloxone in reversing the DXM adverse effects if predominantly opioid in nature led to significantly faster recovery and reduced adverse events, early reversal, and early discharge home. Patients who abuse cough syrup require psychiatric counseling to help nip the inciting factor in the bud. Further research is required to institute the use of naloxone as an antidote for DXM toxicity.
